# Anxiety levels moderate the association between visual acuity and health-related quality of life in chronic eye disease patients

**DOI:** 10.1038/s41598-022-06252-1

**Published:** 2022-02-10

**Authors:** Hugo Senra, Laura Hernandez-Moreno, Natacha Moreno, António Filipe Macedo

**Affiliations:** 1 Centre for Research in Neuropsychology and Cognitive and Behavioural Intervention (CINEICC), University of Coimbra, Coimbra, Portugal; 2 School of Health and Social Care, University of Essex, Colchester, UK; 3 Low Vision and Visual Rehabilitation Lab, Department and Center of Physics—Optometry and Vision Science, University of Minho, Braga, Portugal; 4 Hospital Santa Maria Maior E.P.E, Barcelos, Portugal; 5 Department of Medicine and Optometry, Linnaeus University, Kalmar, Sweden

**Keywords:** Psychology, Diseases, Emotion, Visual system

## Abstract

The current study examines the potential moderating effect of depression and anxiety on the relationship between visual acuity and health-related quality of life in patients with chronic eye diseases. Of the 71 patients, 37 (52%) were female and 34 (48%) were male, age (mean ± SD) was 69 ± 12 years. A significant multivariate regression model was found for patients’ health-related quality of life (EQ-5D-5L index) (R^2^ = 0.43, *p* < 0.001), in which visual acuity (logMAR) (*p* < 0.001), anxiety (HADS-A) (*p* = 0.007), and age of diagnosis (*p* = 0.04)  were independently associated with health-related quality of life (EQ-5D-5L). The moderation model for anxiety (R^2^ = 0.47, F = 5.91, *p* < 0.001) revealed a significant interaction of visual acuity and levels of anxiety in relation to health-related quality of life. Conditional effects analysis suggested that higher logMAR values (which indicate more vision loss) were associated with lower EQ-5D-5L index (indicating worse health-related quality of life), this relationship being stronger (even more negative), when levels of anxiety are high. Clinical and rehabilitation services providing care for chronic eye disease patients should include regular checks for patients’ levels of anxiety, even in patients who still have preserved visual acuity, to help preventing a synergistic source of long-term poor quality of life and disability.

## Introduction

Most cases of visual impairment (65%) and blindness (82%) worldwide occur in people aged 50 years and older^1^. Age-related eye diseases are the leading cause of vision impairment worldwide^1^, such as age-related macular degeneration (AMD), and diabetic retinopathy (DR). Living with DR or AMD can be very challenging for patients, as these conditions cause irreversible and progressive vision loss and visual impairment^1–3^.

Chronic eye diseases, including AMD and DR, are associated with increased risk for mental health problems, particularly depression which is likely to affect 1.59 times more eye disease patients than healthy controls (OR 1.59; 95% CI 1.40–1.81)^4^. Among eye disease patients, depression has an estimated prevalence of 25%, as shown in two meta-analysis^4,5^. Anxiety is also highly prevalent in eye disease patients, with a previous systematic review suggesting a prevalence rate ranging from 9.6 to 30% among AMD patients^6^. A large 5-year longitudinal study evaluated 7584 older adults with self-reported vision impairment and estimated a prevalence of symptoms of depression of 31.2% (95% CI 27.0–35.6%), and a prevalence of symptoms of anxiety of 27.2% (95% CI 23.7–30.9%)^7^. The comorbidity of symptoms of depression and anxiety and eye diseases is deemed to be multifactorial^8–11^. Main factors underlying depression in eye disease patients include reduced distance and near vision^12^, co-morbidities^13^, vision-related disability^7,14,15^, burden of care related to regular hospital visits to receive medical treatments (e.g. anti-VEGF treatment for neovascular AMD)^8^, uncertainty about the prognosis^8^, fear of going blind in the future^8^, poor self-esteem^10^, and poor perceived social support^11^.

Chronic eye diseases, such as AMD and DR, have been associated with an increased risk for deterioration in patients’ quality of life^16–21^. A recent systematic review highlighted a consistent association between vision impairment, eye diseases (age-related macular degeneration, glaucoma, diabetic retinopathy), and reduced quality of life^22^. According to the literature, the main factors contributing to a deterioration in patients’ quality of life included reduced visual acuity^16,18–21,23^, vision-related disability^18,24^, dual sensory impairment (sight and hearing loss)^25^, and other comorbid chronic conditions, including multimorbidity (e.g. diabetes, stroke, rheumatoid arthritis)^20,26,27^. Among these factors, reduced visual acuity was found to be the main responsible factor for poor quality of life in patients with chronic eye diseases^18–21,23^. Progressive vision loss is likely to negatively interfere with daily life activities affecting domains such as reading, mobility, and independence^17,18,28,29^. Other factors potentially affecting patients’ quality of life include economic status and education level^27,29^.

Studies with eye disease patients have used patient‐reported outcome measures to assess patients’ quality of life, including vision-related quality of life measures to address disease-specific domains of quality of life (e.g. Vision Function Questionnaire 25), and health-related quality of life measures to address more general domains of quality of life (e.g. EQ-5D-5L)^16–21,25,28,29^. The regular assessment of patients’ quality of life, together with visual acuity, is key for vision rehabilitation services and eye clinics providing care for chronic eye disease patients^30,31^. The ultimate goal of vision rehabilitation is to reduce the negative impact of vision loss by improving visual ability, i.e., the ability to perform tasks that rely on vision, which is deemed to improve patients vision-related and health-related quality of life^30,31^.

Health-related quality of life in eye disease patients is also associated with adaptation to vision loss which can vary across patients with similar levels of visual impairment^32^. Main factors playing a role in the adaptation to vision loss include coping strategies, vision rehabilitation, and mental health, particularly depression and anxiety^32^. Perceived social support might also play an important role for patents’ coping with vision loss. A recent study conducted with patients with AMD and DR found perceived social support to be independently associated with levels of anxiety and depression, irrespective of patients’ level of visual acuity^11^.

Some factors are, therefore, expected to have a potential moderating effect on the relationship between visual acuity and health-related quality of life in eye disease patients. It is known that mental health problems can be highly comorbid in eye disease patients^4,5^, and that they are likely to deteriorate patients’ health-related quality of life, as depression is in itself an important source of disability^33,34^. However, it is still not clear whether mental health outcomes such as symptoms of depression and anxiety can moderate the relationship between visual acuity and health-related quality of life in eye disease patients. There is extensive and robust literature highlighting a direct and significant association between visual acuity / visual impairment and reduced quality of life in eye disease patients^16–21^, but still very limited evidence on which factors can moderate this relationship.

With the current study we want to examine potential factors moderating the relationship between visual acuity and health-related quality of life in patients with AMD and DR. We expect that lower visual acuity will be significantly associated with poorer health-related quality of life, being this relationship stronger when levels of depression or anxiety are higher. We therefore expect levels of depression or anxiety to moderate the relationship between visual acuity and health-related quality of life, with a potential synergistic effect on that relationship.

## Methods

### Ethics statement

The current study is part of an ongoing clinical trial, which has started in March 2017 (registration number: ISRCTN10894889), addressing the cost-effectiveness of a basic vision rehabilitation service in Portugal. Ethical clearance was granted by the Ethics Committee for Life Sciences and Health of the University of Minho (approval number SECVS 147/2016), and by the Hospital Santa Maria Maior’s ethics committee. The study is registered by the Portuguese data protection authority, with the approval number 7012/2017. All experiments were performed in accordance with relevant guidelines and regulations, and in accordance with the Declaration of Helsinki. Informed consent was obtained from all participants of this study.

### Sample

Eye disease patients attending outpatient appointments at the department of ophthalmology of Hospital Santa Maria Maior E.P.E (Barcelos, Portugal) were invited to attend in-person interviews. Inclusion criteria: primary diagnosis and cause of vision problem DR or AMD; 18 years or older; and living in the community (not any type of assisted living). The exclusion criteria were: cognitive impairment based on scores of mini-mental state examination; communication problems due to, for example, hearing impairment or inability to speak Portuguese; unable to read due to low level of education. For those accepting to take part, demographic and clinic information data including age, gender, educational level, employment status, age of diagnosis and comorbidities was collected. At the time of data collection for this study, none of our patients had started the vision rehabilitation programme. More details on the current study design are available in a previous publication^35^.

### Measures

Presenting distance visual acuity was measured monocularly with ETDRS charts (Early Treatment Diabetic Retinopathy Study), and a letter-by-letter scoring was employed^36,37^. Presenting distance visual acuity was assessed in a dim light room using an internally illuminated cabinet, model 2425E (Precision Vision, IL, USA). Testing distance was adjusted according to the severity of vision loss. ETDRS charts consists of rows of letters, each row comprises 5 letters and white space between letters are equivalent to a letter, each letter corresponds to 0.02 units of acuity and, because of that, letter-by letter scoring can be used. The variable “Visual Acuity” included in our statistical models represents the patients’ presenting distance visual acuity in the better eye, which was measured in logMAR. In this scale, lower values indicate better visual acuity, and higher values indicate worse visual acuity. We adopted the World Health Organization criteria for moderate or severe vision impairment which requires distance visual acuity to be worse than 6/18 (0.477 logMAR)^38^.

Symptoms of anxiety and depression were assessed using the Hospital Anxiety and Depression Scale^39^. Hospital Anxiety and Depression Scale is a self-assessment questionnaire, comprising 2 subscales evaluating levels of depression and levels of anxiety with 7-items each. Each subscale generates scores between 0 and 21, a score of 8 or above indicates the presence of clinically significant levels of anxiety or depression.

Perceived social support was assessed with the Portuguese version of Multidimensional Scale of Perceived Social Support^40^. This scale has 12 questions that are divided into 3 subcategories (family, friends and significant others) with 4 questions each. The higher the score, the better is the perceived social support.

Health-related quality of life was assessed using the EQ-5D-5L. This instrument was developed by the EuroQol group^41^. This instrument assessed 5 dimensions of quality of life, including mobility, self-care, usual activities, pain/discomfort and anxiety/depression. Each dimension has five possible levels of response: no problems, slight, moderate, severe or unable to undertake the particular action. A standardized value set is obtained from the 5 dimensions, with higher scores indicating better quality of life.

Demographic and clinical data were collected using a questionnaire and via patients’ medical records.

### Data analysis

In the current study, we aimed to run regression models, with a maximum of 10 predictors included in a single model, namely, presenting distance visual acuity, anxiety, depression, perceived social support, age of diagnosis (up to 1 year; more than 1 year), comorbidities (up to 1 vs 2 or more), employment status (full time work vs retirement), education level (≤ 9 years vs > 9 years of education), age, and gender (male vs female), as these variables are potential factors of health-related quality of life for eye disease patients^27,29^. Criteria for categorizing clinical variables included: recent vs non-recent diagnosis (age of diagnosis); presence or absence of multimorbidity (2 or more conditions for comorbidities). G-Power software^42^ was used to compute the minimum sample size required for a multiple regression model including 10 predictors. With type I error rate (alpha) set at 0.05 (two-tailed), and aiming to a power of 0.95, an effect size of 0.5, and a critic F of 2.03, a minimum of 59 participants is required.

Data analysis was performed with SPSS (IBM SPSS Statistics for Windows, Version 27.0. Armonk, NY: IBM Corp). Demographic (age, gender, education, employment status) and clinical-related variables (diagnosis, age of diagnosis, comorbidities, visual acuity, depression, anxiety, perceived social support) were summarized for the whole sample. Depression and anxiety variables were square root transformed to reduce right skewness and meet the assumption of normal distribution of residuals for the regression models included in our study. Mann–Whitney-U test was used to investigate differences in quality of life scores for gender, education, group (presence or absence of vision impairment), comorbidities, and diagnostic type (age-related macular degeneration, or diabetic retinopathy).

Multivariate regression analysis was run to identify independent factors of health-related quality of life within our sample, considering findings from previous studies^16–21,27,29^. The regression model included the 10 predictors mentioned above. Regression diagnostics were conducted and no outliers were detected. Model’s variance inflation factor values ranged between 1.10 and 3.30, and the tolerance ranged between 0.28 and 0.87, indicating no collinearity among predictors. Assumptions of normality for residuals and homogeneity of variance were met.

**Figure 1:**
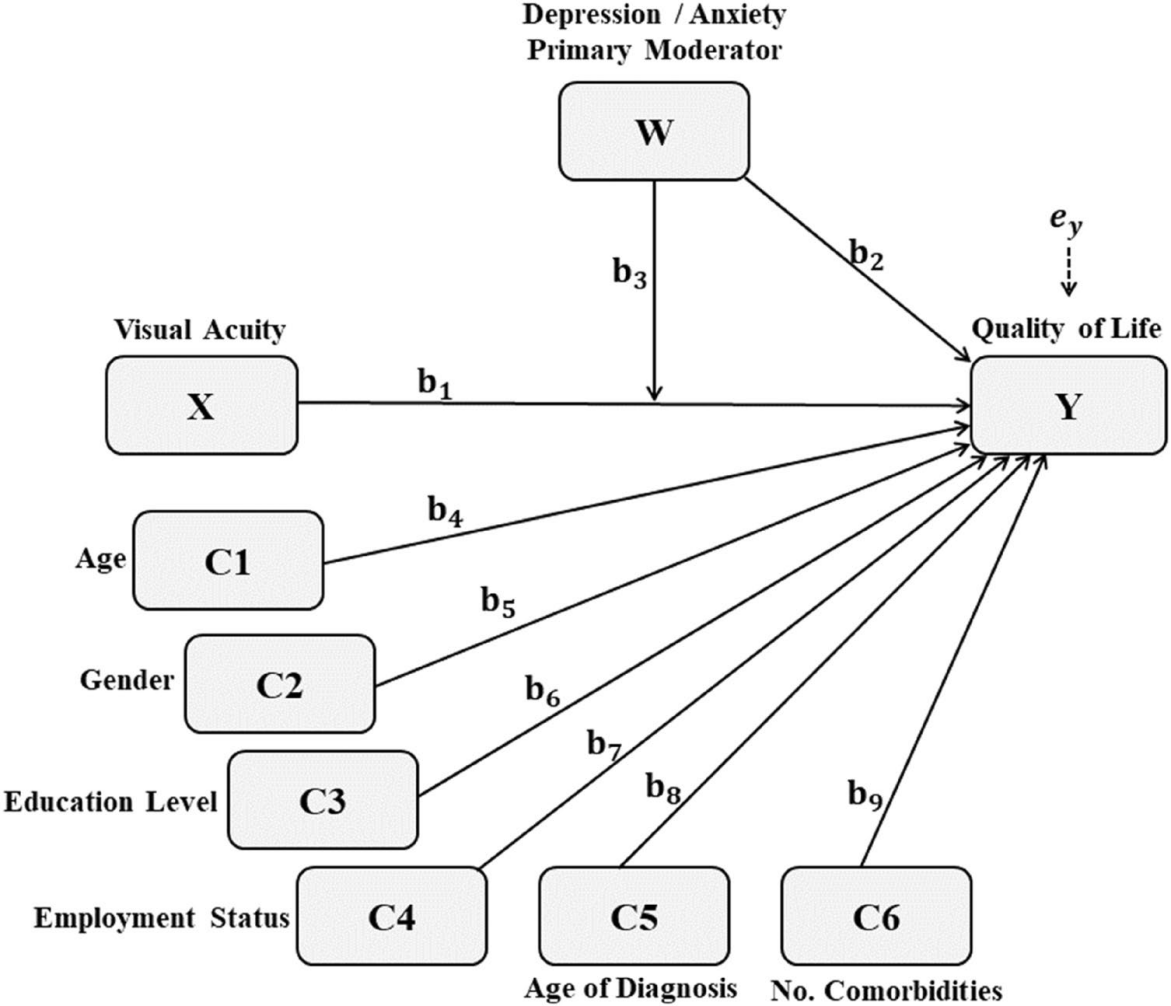
Moderation model for the relationship between visual acuity and quality of life, with anxiety or depression as moderators, and age, gender, education level, employment status, age of diagnosis and number of comorbidities as covariates.

Stepwise moderation models were performed to assess whether depression and anxiety were significant moderators of the relationship between presenting distance visual acuity in the better seeing eye (using a continuous acuity logMAR scale) and health-related quality of life (EQ-5D-5L index). For each model predicting patients’ health-related quality of life, we assessed the predictive effect of visual acuity, under the effect of a primary moderator, depression or anxiety, and controlling for potential confounders such as age, gender, education level, employment status, age of diagnosis and number of comorbidities. Highest order unconditional interaction effect was assessed to investigate whether anxiety or depression (W) were significant moderators of the relationship between visual acuity (X) and health-related quality of life (Y). The conditional effects were examined in both models, in which the moderation of X’s effect (visual acuity) on Y (health-related quality of life) by W (depression / anxiety) is tested, while controlling for age (C1), gender (C2), education level (C3), employment status (C4), age of diagnosis (C5), and number of comorbidities (C6), as given in the following equation and in Fig. 1.

$$Y = i_{Y} + b_{1} X + b_{2} W + b_{3} XW + b_{4} C1 + b_{5} C2 + b_{6} C3 + b_{7} C4 + b_{8} C5 + b_{9} C6 + e_{Y}$$

X and W variables were mean centred for all moderation models. All variables included in the moderation models were continuous, except for gender, education level, employment status, age of diagnosis, and number of comorbidities.

Sensitivity analysis was computed to investigate an alternative moderation model with perceived social support as a secondary moderator (having anxiety or depression as primary moderator), as previous literature suggested that social support is independently associated with depression and anxiety in eye disease patients^11^. This model tested the hypothesis that social support would moderate the moderation effect of depression or anxiety on the relationship between visual acuity and health-related quality of life (Supplementary Figure S1). Moderation analysis was run using the PROCESS module in SPSS^43^.

## Results

Seventy-one patients (142 eyes) with AMD or DR participated in this study. All patients presented other medical comorbidities with 70% of patients (N = 50) presenting multimorbidity. With the exception of AMD, DR, and diabetes, high blood pressure (N = 51), musculoskeletal disorders (N = 25) and cardiovascular disease (N = 13) were the most frequent comorbid health problems. Sample characteristics are presented in Table 1, and group comparisons for patients characteristics in relation to health-related quality of life (EQ-5D-5L index) are presented in Table 2.

**Table 1:** Demographic and clinical data.

N = 71	Mean (SD)
Age	68.80 (11.96)
Age of Diagnosis (years)	3.70 (3.28)
Presenting Distance Visual Acuity (LogMar)	0.42 (0.33)
Health-related quality of life (EQ-5D-5L)	0.82 (0.20)
Anxiety (HADS-A*)	4.32 (3.82)
Depression (HADS-D*)	4.41 (3.39)
Perceived social support (MSPSS)	5.29 (0.61)

*EQ-5D-5L* EuroQol Questionnaire of health-related quality of life, *HADS* Hospital Anxiety and Depression Scale, *MSPSS* Multidimensional Scale of Perceived Social Support.

*Raw scores.

**Table 2:** Group comparisons for sample characteristics.

N = 71		Frequency (%)	EQ-5D quality of life^a^	*p* value	Effect size (r)
Gender	Female	37 (52%)	30.74	0.02	0.27
	Male	34 (48%)	41.72		
Education	≤ 9 years	50 (70%)	33.48	0.10	NS
	> 9 years	21 (30%)	42.00		
Employment status	Full-time work	11 (15.5%)	51.23	0.006	0.32
	Retirement	60 (84.5%)	33.21		
Vision impairment*	No vision impairment	40 (56.3%)	44.88	< 0.001	0.50
	Moderate or Severe Vision Impairment	31 (43.7%)	24.55		
Comorbidities	Up to 1	21 (29.5%)	38.57	0.485	NS
	2 or more	50 (70.5%)	34.92		
Diagnosis	AMD	18 (25%)	38.89	0.038	0.25
	DR	53 (75%)	27.50		
Age of diagnosis	Up to 1 year (recent)	20 (28%)	30.43	0.144	NS
	More than 1 year	51 (72%)	38.19		

*AMD* age-related macular degeneration, *DR* diabetic retinopathy, *NS* Not significant.

*World Health Organization criteria for moderate or severe vision impairment which requires distance visual acuity to be worse than 6/18 or 0.477 logMAR.

^a^Mean ranks obtained from Mann–Whitney U test.

Multivariate regression analysis (Table 3) led to a final significant model explaining 43% of the variance in patients’ health-related quality of life (EQ-5D-5L index) (R^2^ = 0.43, *p* < 0.001), in which visual acuity (*p* < 0.001), anxiety (*p* = 0.007), and age of diagnosis (*p* = 0.04) were independently associated with health-related quality of life. Worse visual acuity, higher levels of anxiety and an older diagnosis were significantly associated with poorer quality of life.

**Table 3:** Multivariate regression analysis to identify independent factors of patients’ health-related quality of life.

N = 71	Unstandardized coefficient (SE)
Visual acuity^a^	− 0.32 (0.07)*
Anxiety^a^	− 0.10 (0.04)**
Depression^a^	0.06 (0.04)
Social support^a^	0.01 (0.04)
Age of diagnosis (0 to 1 year vs > 1 year)	0.10 (0.05)**
Comorbidities (0 to 1 vs > 1)	− 0.04 (0.05)
Employment status (Full-time work vs retirement)	− 0.03 (0.08)
Age^a^	0.00 (0.00)
Gender (F, M)	− 0.04 (0.04)
Education level (up to 9 years vs > 9 years)	0.05 (0.05)

*F* female, *M* male.

*p < 0.001; **p < 0.05.

^a^Continuous variables.

**Figure 2:**
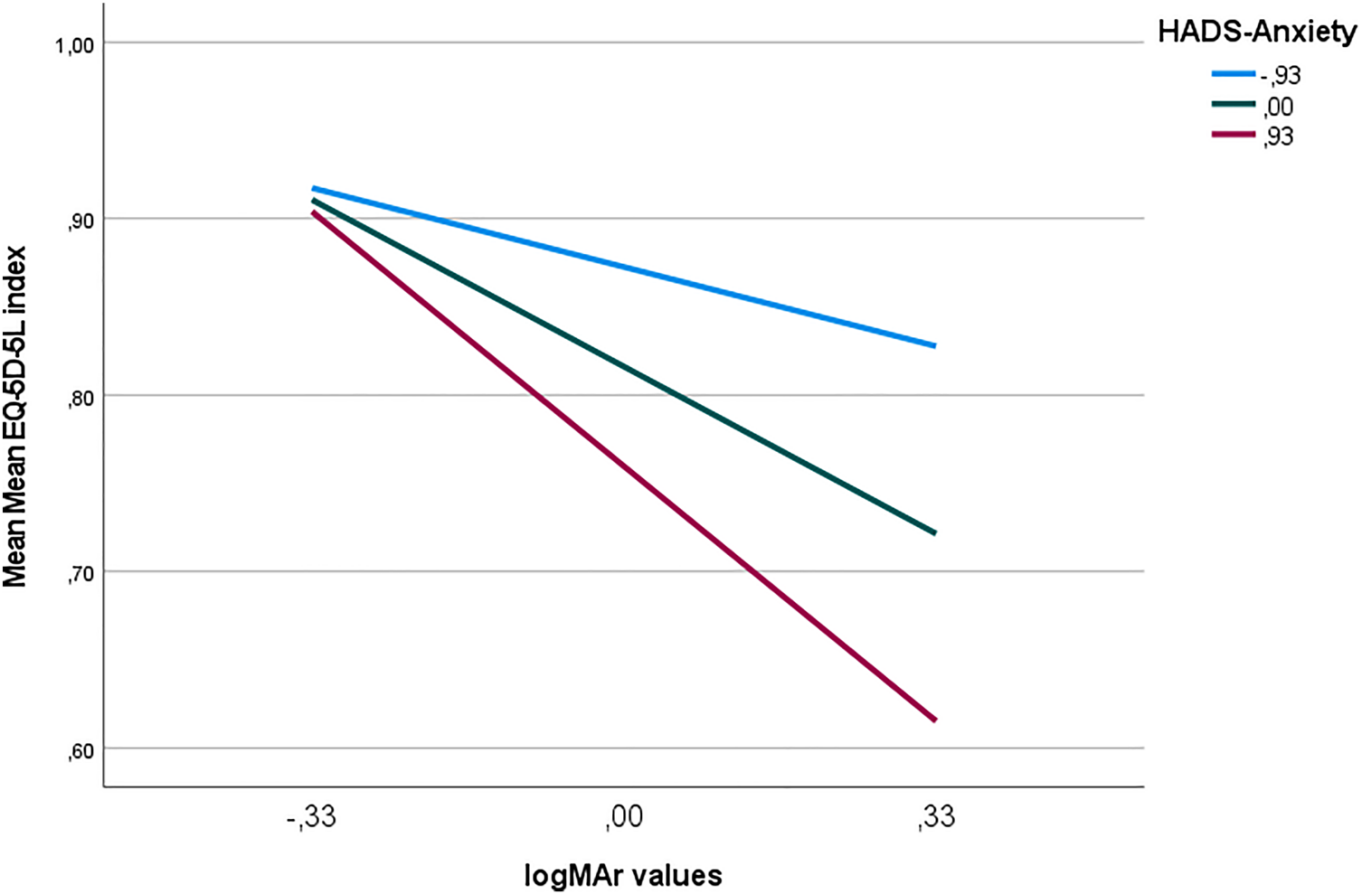
Results of the significant moderation model for the conditional relationship between Visual Acuity (logMAR values) and Health-related Quality of Life (EQ-5D-5L index), moderated by anxiety (HADS-A scores). Higher logMAR values mean worse visual acuity. The light blue regression line shows the relationship between EQ-5D-5L index and logMAR values at 1 SD below the mean on HADS-Anxiety (− 0.93). The dark blue line shows the relationship between EQ-5D-5L index and logMAR values at the mean on HADS-Anxiety (0.00). The red line shows the relationship between EQ-5D-5L index and logMAR values at 1 SD above the mean on HADS-Anxiety (0.93).

**Figure 3:**
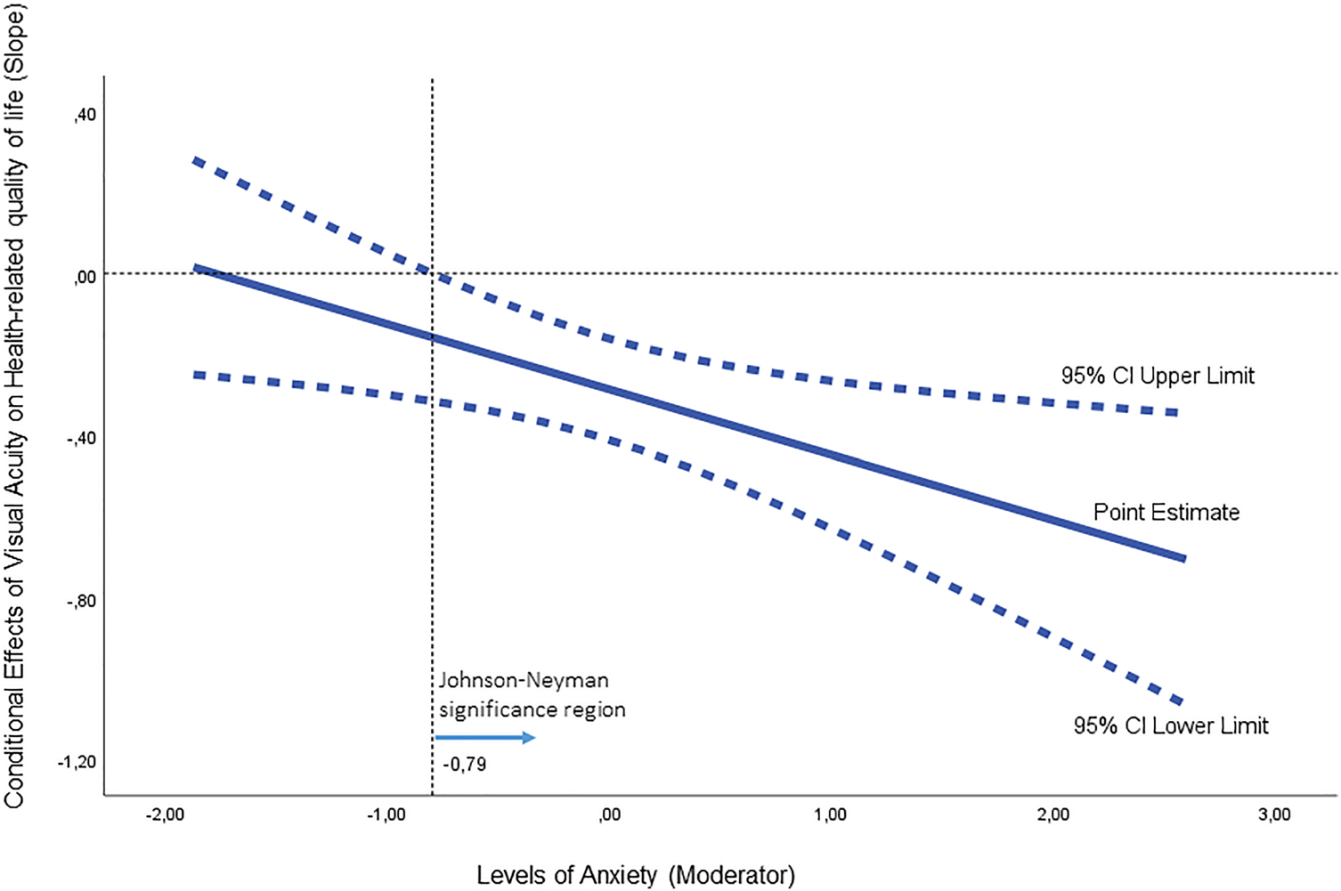
Johnson-Neyman plot depicting conditional effects of visual acuity on change within health-related quality of life at values of the moderator anxiety. The range of observed values of anxiety is [− 0.93, 0.93].

Two stepwise moderator models were run to examine the potential moderating effect of anxiety and depression on the relationship between visual acuity and health-related quality of life, while controlling for age, gender, education level, employment status, number of comorbidities, and age of diagnosis (covariates in both models). The moderation model for anxiety (R^2^ = 0.47, F = 5.91, *p* < 0.001) revealed a significant interaction of visual acuity and levels of anxiety in relation to health-related quality of life (Tables 4, 5). Age of diagnosis was also significantly and independently associated with health-related quality of life, with more years of diagnosis being associated with better quality of life outcomes. Conditional effects analysis revealed a negative relationship between logMAR values of visual acuity and the EQ-5D-5L index, which is positively moderated by HADS-A scores (levels of anxiety). Higher logMAR values (which indicate greater vision loss) were associated with lower EQ-5D-5L index values (indicating poorer health-related quality of life), being this relationship stronger (even more negative), when levels of anxiety are high (Table 5, Figs. 2, 3). The moderation model for depression (R^2^ = 0.37, F = 4.03, *p* < 0.001) revealed a non-significant interaction between visual acuity and levels of depression in relation to health-related quality of life (Table 6).

**Table 4:** Stepwise moderation analysis with anxiety (W) as moderator of the relationship between visual acuity (X) and quality of life (Y).

N = 71			Coefficient	SE	t	*p*
Model 1 R^2^ = 0.26, MSE = 0.17 R^2^ change = 0.26	Constant	i_y_	0.95	0.03	28.30	< 0.001
	Visual acuity* (X)	b_1_	− 0.31	0.06	− 4.94	< 0.001
Model 2 R^2^ = 0.35, MSE = 0.16 R^2^ change = 0.35	Constant	i_y_	1.07	0.05	20.72	< 0.001
	Visual acuity (X)	b_1_	− 0.32	0.06	− 5.34	< 0.001
	Anxiety (W)	b_2_	− 0.06	0.02	− 2.98	0.004
Model 3 R^2^ = 0.41, MSE = 0.03 R^2^ change = 0.06	Constant	i_y_	0.82	0.02	43.53	< 0.001
	Visual acuity (X)	b_1_	− 0.32	0.06	− 5.59	< 0.001
	Anxiety (W)	b_2_	− 0.06	0.02	− 3.16	0.002
	XW	b_3_	− 0.17	0.06	− 2.70	0.009
Model 4 (Final Model) R^2^ = 0.47, MSE = 0.02 R^2^ change = 0.06	Constant	i_y_	0.79	0.14	5.54	< 0.001
	Visual acuity (X)	b_1_	− 0.29	0.06	− 4.46	< 0.001
	Anxiety (W)	b_2_	− 0.06	0.02	− 2.67	0.009
	XW	b_3_	− 0.16	0.06	− 2.52	0.014
	Age (C1)	b_4_	− 0.04	0.04	− 0.86	0.388
	Gender (C2)	b_5_	0.00	0.00	0.03	0.974
	Education level (C3)	b_6_	0.04	0.05	0.88	0.380
	Employment status (C4)	b_7_	− 0.01	0.07	− 0.19	0.846
	Age of diagnosis (C5)	b_8_	0.09	0.04	2.02	0.047
	Number of comorbidities (C6)	b_9_	− 0.03	0.04	− 0.75	0.453

*Presenting distance visual acuity in the better eye (logMar values); categorical variables: gender (female; male); education level (up to 9 years of education; more than 9 years of education); employment status (full-time work; retirement); age of diagnosis (up to 1 year; more than 1 year); number of comorbidities (up to 1; 2 or more).

**Table 5:** Unconditional and conditional effects for the moderating effect of anxiety on the relationship between visual acuity and health-related quality of life.

Moderation model (X and Y variables)		Highest order unconditional interaction effect (visual acuity x anxiety)			Conditional effects of visual acuity at values of anxiety (mean centred)		
		R^2^ change	F	*p*-value	Lowest level (− 0.93) t-statistic (*p*-value)	Moderate level (0.00) t-statistic (*p*-value)	Highest level (0.93) t-statistic (*p*-value)
Visual Acuity (logMAR values)	Health-related Quality of life (EQ-5D-5L index)	.060	6.35	0.01	− 1.06 (0.11)	− 4.06 (< 0.001)*	− 4.96 (< 0.001)*

Moderation model examining levels of anxiety as a moderator of the relationship between visual acuity and quality of life (highest order unconditional interactions). The conditional effects of visual acuity were examined at different levels of anxiety to determine if the relationship between visual acuity and quality of life was different in patients presenting low, moderate, or high levels of anxiety. *p-value < 0.05.

**Table 6:** Stepwise moderation analysis with depression (W) as moderator of the relationship between visual acuity (X) and health-related quality of life (Y).

N = 71			Coefficient	SE	t	*p*
Model 1 R^2^ = 0.26, MSE = 0.17 R^2^ change = 0.26	Constant	i_y_	0.95	0.03	28.29	< 0.001
	Visual acuity* (X)	b_1_	− 0.31	0.06	− 4.94	< 0.001
Model 2 R^2^ = 0.29, MSE = 0.17 R^2^ change = 0.29	Constant	i_y_	1.02	0.05	18.77	< 0.001
	Visual acuity (X)	b_1_	− 0.29	0.06	− 4.46	< 0.001
	Depression (W)	b_2_	− 0.04	0.03	− 1.59	0.117
Model 3 R^2^ = 0.31, MSE = 0.03 R^2^ change = 0.03	Constant	i_y_	0.83	0.02	39.63	< 0.001
	Visual acuity (X)	b_1_	− 0.29	0.06	− 4.61	< 0.001
	Depression (W)	b_2_	− 0.04	0.02	− 1.78	0.079
	XW	b_3_	− 0.12	0.08	− 1.59	0.116
Model 4 (Final model) R^2^ = 0.37, MSE = 0.03 R^2^ change = 0.02	Constant	i_y_	0.76	0.15	4.95	< 0.001
	Visual acuity (X)	b_1_	− 0.26	0.07	− 3.82	0.003
	Depression (W)	b_2_	− 0.03	0.03	− 0.96	0.338
	XW	b_3_	− .010	0.08	− 1.27	0.208
	Age (C1)	b_4_	0.00	0.00	0.49	0.625
	Gender (C2)	b_5_	− 0.05	0.04	− 1.19	0.237
	Education level (C3)	b_6_	0.05	0.05	0.93	0.356
	Employment STATUS (C4)	b_7_	− 0.05	0.08	− 0.62	0.535
	Age of Diagnosis (C5)	b_8_	0.08	0.05	1.17	0.972
	Number of comorbidities (C6)	b_9_	− 0.03	0.05	− 0.61	0.544

*Presenting distance visual acuity in the better eye (logMar values); categorical variables: gender (female; male); education level (up to 9 years of education; more than 9 years of education); employment status (Full-time work; retirement); age of diagnosis (up to 1 year; more than 1 year); number of comorbidities (up to 1; 2 or more).

Sensitivity analysis showed a non-significant interaction between visual acuity and our primary moderators (anxiety or depression) under the effect of the secondary moderator (perceived social support) (see Supplementary Table S1).

## Discussion

In this study we investigated factors independently associated with health-related quality of life in eye disease patients. In particular, we tested the hypothesis that mental health outcomes, anxiety and depression, moderate the relationship between visual acuity and health-related quality of life in patients with DR and AMD. Our findings partially corroborate this hypothesis because anxiety, but not depression, significantly and positively moderated the relationship between visual acuity and health-related quality of life.

In our model, anxiety has a moderating and synergistic effect on the relationship between visual acuity and health-related quality of life. When patients’ levels of anxiety are higher, the negative effect that reduced visual acuity has on health-related quality of life is expected to be stronger. This is consistent with the current state of the art which has highlighted the potential negative and disabling effect of comorbid mental health problems on eye disease patients’ quality of life^21,44,45^. Furthermore, previous studies have identified common factors of mental health problems among eye disease patients, which help us to understand why anxiety levels might play a key role in patients’ health-related quality of life. These factors include patients’ experiences of anticipatory anxiety related to medical treatments and prognosis, such as the fear of going blind in the future, fear of intravitreal injections received on a regular basis for neovascular AMD, uncertainty about disease prognosis, burden related to regular hospital visits, and experiences of loneliness^8–11^. These factors tend to be recurrent among chronic eye disease patients, and have in common their potential for increasing patients’ levels of anxiety, which would ultimately contribute to deteriorate their health-related quality of life.

Coping with vision loss has been described as an emotionally distressing and challenging process^32^. Previous studies have highlighted the potential benefits of a good perceived social support^11^, acceptance as a coping strategy^32^, and an effective vision rehabilitation^30–32^. Patients with an older diagnosis are therefore more likely to have received some support for vision loss such as vision rehabilitation, which can explain why in our sample patients with older diagnosis showed slightly better quality of life outcomes.

Previous literature has highlighted the potential long-term disabling effect of anxiety disorders^46,47^. The World Health Organization ranked anxiety disorders as the sixth largest contributor to global disability, having accounted for 3.4% of all years lived with disability worldwide, and 4.2% in high-income countries^47^. It is known that anxiety is highly prevalent among chronic eye disease patients^6,7^, which encompasses an additional source of disability and reduced quality of life for these patients. This is of paramount importance as previous studies have revealed that anxiety and depression in chronic eye disease patients tend to be neglected by health services, and consequently many patients remain untreated for their mental health and well-being needs^8,48–51^.

The fact that in our moderation model depression was not found to be a significant moderator of the relationship between visual acuity and health-related quality of life is intriguing. This finding goes against the previous literature suggesting that depression has an eminent negative effect on patients’ quality of life^21,26,27^, and that it is in itself a potential source of long-term disability^33,34^. A possible explanation for this finding is the fact that our sample size was relatively small to allow us to detect a significant effect for depression. On a larger sample of eye disease patients, we could expect less statistical noise and the effect of depression on patients’ quality of life to be stronger, as suggested by previous studies^4,5,8^. Another plausible hypothesis to explain these findings is the possible indirect effect of visual acuity on depression. Recent studies have suggested that visual acuity might not be directly and significantly associated with depression, with other factors such as self-esteem, and illness perceptions, being likely to play a more triggering role for the occurrence of depression among these patients^8,10,11^. This hypothesis would explain why depression failed to moderate the relationship between visual acuity and health-related quality of life, as in our model we did not account for the potential moderating effect of those other factors. The absence of a significant interaction effect between visual acuity and depression on patients’ health-related quality of life might also be related to the outcome measure used to assess levels of depression, in our study, the HADS. The HADS has been extensively used in studies with eye disease patients and is regarded a validated measure of depression for this clinical groups^4–6,8^. However, studies with patients with other chronic medical conditions such as cancer^52^ and chronic obstructive pulmonary disease^53^ have suggested that the HADS-D exhibits low diagnostic accuracy for depression. Further research will confirm the actual diagnostic accuracy of HADS for eye disease patients but this hypothesis might also explain our findings for depression. Finally, future studies will clarify the actual importance of depression as a moderator between visual acuity and health-related quality of life, and whether other factors are of statistical importance for such a moderating model.

The main strengths of this study include: (1) a sample composed of patients with different levels of visual acuity which allowed us to examine the potential relationship between visual acuity and quality of life outcomes; (2) the use of robust and widely validated outcome measures, such as the EQ-5D-5L and the HADS; and (3) the novelty of our findings, considering the paucity of studies examining potential factors of poor quality of life, and significant moderators of the relationship between visual acuity and quality of life, among eye disease patients. Main limitations in our study include the size of the sample and the lack of control group (people without any eye diseases). The lack of a control group limits the extent of our findings because people without age-related macular degeneration and diabetic retinopathy, in particular older patients, are also likely to present quality of life problems. Additionally, we did not collect patients’ data on severity of comorbidities which would be an important confounder to be included in our regression models. Finally, in our study the variable “visual acuity” was assessed in relation to presenting distance visual acuity, and some previous studies have suggested that binocular visual acuity might be a better estimate of vision-related quality of life compared with monocular visual acuity^54,55^. The investigation of the effect binocular visual acuity on patients’ quality of life accounting for mental health and psychosocial outcomes should therefore deserve more attention in future studies.

In summary, our study suggests that high levels of anxiety together with low levels of visual acuity can have a potential negative synergistic effect on eye disease patients’ health-related quality of life. Future studies should clarify the actual importance of depression and other key mental health outcome for the relationship between visual acuity and health-related quality of life. Clinical and rehabilitation services providing care for chronic eye disease patients should include regular checks for patients’ levels of anxiety, even in patients who still have preserved visual acuity, above the threshold of what is considered visual impairment. This will contribute to prevent a synergistic source of long-term poor quality of life and disability, in a particularly vulnerable patient group already affected by a chronic condition.

## Supplementary Information

Supplementary Information.

## References

[1] Stevens GA, White RA, Flaxman SR (2013). Global prevalence of vision impairment and blindness: Magnitude and temporal trends, 1990–2010. Ophthalmology.

[2] Taylor DJ, Hobby AE, Binns AM, Crabb DP (2016). How does age-related macular degeneration affect real-world visual ability and quality of life? A systematic review. BMJ Open.

[3] Nagda D, Mitchell W, Zebardast N (2021). The functional burden of diabetic retinopathy in the United States. Graefes Arch Clin Exp Ophthalmol..

[4] Zheng Y, Wu X, Lin X, Lin H (2017). The prevalence of depression and depressive symptoms among eye disease patients: A systematic review and meta-analysis. Sci. Rep..

[5] Parravano M, Petri D, Maurutto E (2021). Association between visual impairment and depression in patients attending eye clinics: A meta-analysis. JAMA Ophthalmol..

[6] Dawson SR, Mallen CD, Gouldstone MB, Yarham R, Mansell G (2014). The prevalence of anxiety and depression in people with age-related macular degeneration: A systematic review of observational study data. BMC Ophthalmol..

[7] Frank CR, Xiang X, Stagg BC, Ehrlich JR (2019). Longitudinal associations of self-reported vision impairment with symptoms of anxiety and depression among older adults in the United States. JAMA Ophthalmol..

[8] Senra H, Balaskas K, Mahmoodi N (2017). Experience of anti-VEGF treatment and clinical levels of depression and anxiety in patients with wet age-related macular degeneration. Am. J. Ophthalmol..

[9] Grant A, Aubin MJ, Buhrmann R (2021). Visual impairment, eye disease, and the 3-year incidence of depressive symptoms: The Canadian longitudinal study on aging. Ophthalmic Epidemiol..

[10] Maaswinkel IM, van der Aa HPA, van Rens GHMB (2020). Mastery and self-esteem mediate the association between visual acuity and mental health: A population-based longitudinal cohort study. BMC Psychiatry.

[11] Hernández-Moreno L, Senra H, Moreno N (2021). Is perceived social support more important than visual acuity for clinical depression and anxiety in patients with age-related macular degeneration and diabetic retinopathy?. Clin. Rehabil..

[12] Zhang X, Bullard KM, Cotch MF, Wilson MR, Rovner BW, McGwin G, Owsley C, Barker L, Crews JE, Saaddine JB (2013). Association between depression and functional vision loss in persons 20 years of age or older in the United States, NHANES 2005–2008. JAMA Ophthalmol..

[13] Langelaan M, de Boer MR, van Nispen RM, Wouters B, Moll AC, van Rens GH (2007). Impact of visual impairment on quality of life: A comparison with quality of life in the general population and with other chronic conditions. Ophthalmic Epidemiol..

[14] Choi HG, Lee MJ, Lee SM (2018). Visual impairment and risk of depression: A longitudinal follow-up study using a national sample cohort. Sci. Rep..

[15] Nollett C, Ryan B, Bray N (2019). Depressive symptoms in people with vision impairment: A cross-sectional study to identify who is most at risk. BMJ Open.

[16] Nickels S, Schuster AK, Elflein H, Wolfram C, Schulz A, Münzel T, Beutel ME, Schmidtmann I, Finger RP, Pfeiffer N (2019). Vision-related quality of life considering both eyes: Results from the German population-based Gutenberg Health Study (GHS). Health Qual. Life Outcomes..

[17] Man REK, Gan ATL, Fenwick EK, Gupta P, Thakur S, Fang XL, Cheng CY, Wong TY, Lamoureux EL (2021). The differential impact of age on vision-related quality of life across the visual impairment spectrum. Ophthalmology.

[18] Crews JE, Chou CF, Zack MM, Zhang X, Bullard KM, Morse AR, Saaddine JB (2016). The association of health-related quality of life with severity of visual impairment among people aged 40–64 years: Findings from the 2006–2010 behavioral risk factor surveillance system. Ophthalmic Epidemiol..

[19] De Sousa PR, Krstic L, Hill SCL, Foss AJE (2021). Predicting quality of life in AMD patients-insights on the new NICE classification and on a bolt-on vision dimension for the EQ-5D. Eye (Lond)..

[20] Taipale J, Mikhailova A, Ojamo M (2019). Low vision status and declining vision decrease Health-Related Quality of Life: Results from a nationwide 11-year follow-up study. Qual. Life Res..

[21] Purola PKM, Nättinen JE, Ojamo MUI (2021). Prevalence and 11-year incidence of common eye diseases and their relation to health-related quality of life, mental health, and visual impairment. Qual. Life Res..

[22] Assi L, Chamseddine F, Ibrahim P, Sabbagh H, Rosman L, Congdon N, Evans J, Ramke J, Kuper H, Burton MJ, Ehrlich JR, Swenor BK (2021). A global assessment of eye health and quality of life: A systematic review of systematic reviews. JAMA Ophthalmol..

[23] Nakano T, Kawashima M, Hiratsuka Y, Tamura H, Ono K, Murakami A, Tsubota K, Yamada M (2016). Assessment of quality of life in patients with visual impairments using a new visual function questionnaire: The VFQ-J11. Clin. Ophthalmol..

[24] Taylor DJ, Hobby AE, Binns AM, Crabb DP (2016). How does age-related macular degeneration affect real-world visual ability and quality of life? A systematic review. BMJ Open.

[25] Tseng YC, Liu SH, Lou MF, Huang GS (2018). Quality of life in older adults with sensory impairments: A systematic review. Qual. Life Res..

[26] Park SJ, Ahn S, Woo SJ, Park KH (2015). Extent of exacerbation of chronic health conditions by visual impairment in terms of health-related quality of life. JAMA Ophthalmol..

[27] Pan CW, Cong XL, Zhou HJ, Wang XZ, Sun HP, Xu Y, Wang P (2018). Evaluating health-related quality of life impact of chronic conditions among older adults from a rural town in Suzhou, China. Arch. Gerontol. Geriatr..

[28] Pondorfer SG, Terheyden JH, Heinemann M, Wintergerst MWM, Holz FG, Finger RP (2019). Association of vision-related quality of life with visual function in age-related macular degeneration. Sci. Rep..

[29] Schliermann R, Heydenreich P, Bungter T, Anneken V (2017). Health-related quality of life in working-age adults with visual impairments in Germany. Disabil. Rehabil..

[30] Stelmack JA (2017). Outcomes of the veterans affairs Low Vision Intervention Trial II (LOVIT II): A randomized clinical trial. JAMA Ophthalmol..

[31] Binns AM (2012). How effective is low vision service provision? A systematic review. Surv. Ophthalmol..

[32] Senra H, Barbosa F, Ferreira P, Vieira CR, Perrin PB, Rogers H, Rivera D, Leal I (2015). Psychologic adjustment to irreversible vision loss in adults: A systematic review. Ophthalmology.

[33] Christman S, Bermudez C, Hao L, Landman BA, Boyd B, Albert K, Woodward N, Shokouhi S, Vega J, Andrews P, Taylor WD (2020). Accelerated brain aging predicts impaired cognitive performance and greater disability in geriatric but not midlife adult depression. Transl. Psychiatry..

[34] Behrens-Wittenberg E, Wedegaertner F (2020). Identifying individuals at high risk for permanent disability from depression and anxiety. Front. Psychiatry..

[35] Hernández-Moreno L, Senra H, Lewis P, Moreno N, Linhares J, Santana R, Ramos PL, Marques AP, Macedo AF (2020). Cost-effectiveness of basic vision rehabilitation (The basic VRS-effect study): Study protocol for a randomised controlled trial. Ophthalmic Physiol. Opt..

[36] Ferris FL, Kassoff A, Bresnick GH, Bailey I (1982). New visual-acuity charts for clinical research. Am. J. Ophthalmol..

[37] Macedo AF, Ramos PL, Hernandez-Moreno L, Cima J, Baptista AMG, Marques AP (2017). Visual and health outcomes, measured with the activity inventory and the EQ-5D, in visual impairment. Acta Ophthalmol..

[38] International Classification of Diseases. ICD-11 for mortality and morbidity statistics (ICD-11 MMS). https://icd.who.int/browse11/l-m/en. Accessed 20 Sept 2021.

[39] Pais-Ribeiro J, Silva I, Ferreira T (2007). Validation study of a Portuguese version of the Hospital Anxiety and Depression Scale. Psychol. Health Med..

[40] Carvalho S, Pinto-Gouveia J, Pimentel P, Maia D, Mota-Pereira J (2011). Características psicométricas da versão portuguesa da Escala Multidimensional de Suporte Social Percebido (Multidimensional Scale of Perceived Social Support—MSPSS). Psychologica..

[41] Ferreira PL, Antunes P, Ferreira LN, Pereira LN, Ramos-Goñi JM (2019). A hybrid modelling approach for eliciting health state preferences: The Portuguese EQ-5D-5L value set. Qual. Life Res..

[42] Faul F, Erdfelder E, Buchner A, Lang A-G (2009). Statistical power analyses using G*Power 3.1: Tests for correlation and regression analyses. Behav. Res. Methods.

[43] Hayes A (2018). Introduction to Mediation, Moderation, and Conditional Process Analysis. A Regression-Based Approach.

[44] Bian W, Wan J, Tan M, Su J, Yuan Y, Wang Z, Li S (2020). Predictors of health-related quality of life in Chinese patients receiving treatment for neovascular age-related macular degeneration: A prospective longitudinal study. BMC Ophthalmol..

[45] Li Y, Crews JE, Elam-Evans LD, Fan AZ, Zhang X, Elliott AF, Balluz L (2011). Visual impairment and health-related quality of life among elderly adults with age-related eye diseases. Qual. Life Res..

[46] Hendriks SM, Spijker J, Licht CM (2016). Long-term disability in anxiety disorders. BMC Psychiatry.

[47] World Health Organization (2017). Depression and Other Common Mental Disorders: Global Health Estimates.

[48] Holloway EE, Sturrock BA, Lamoureux EL, Keeffe JE, Rees G (2015). Help seeking among vision-impaired adults referred to their GP for depressive symptoms: Patient characteristics and outcomes associated with referral uptake. Aust. J. Prim. Health.

[49] Nyman SR, Dibb B, Victor CR, Gosney MA (2012). Emotional well-being and adjustment to vision loss in later life: A meta-synthesis of qualitative studies. Disabil. Rehabil..

[50] Rees G, Tee HW, Marella M (2010). Vision-specific distress and depressive symptoms in people with vision impairment. Invest. Ophthalmol. Vis. Sci..

[51] Rees G, Ponczek E, Hassell JB, Keeffe J, Lamoureux E (2010). Psychological outcomes following interventions for people with low vision: A systematic review. Expert Rev. Ophthalmol..

[52] Morse R, Kendell K, Barton S (2005). Screening for depression in people with cancer: The accuracy of the hospital anxiety and depression scale. Clin. Eff. Nurs..

[53] Nowak C, Sievi NA, Clarenbach CF, Schwarz EI, Schlatzer C, Brack T, Brutsche M, Frey M, Irani S, Leuppi JD, Rüdiger J, Thurnheer R, Kohler M (2014). Accuracy of the Hospital Anxiety and Depression Scale for identifying depression in chronic obstructive pulmonary disease patients. Pulm. Med..

[54] Man REK, Gan ATL, Fenwick EK, Thakur S, Gupta P, Teo ZL, Cheng CY, Wong TY, Lamoureux EL (2020). Using uniocular visual acuity substantially underestimates the impact of visual impairment on quality of life compared with binocular visual acuity. Ophthalmology.

[55] Liao KM, Wu WC, Jang Y (2021). Impacts of monocular, binocular, and functional visual acuity on vision-related quality of life in patients with type 2 diabetes. Sci. Rep..

