# Supplementary material for: Anxiety levels moderate the association between visual acuity and health-related quality of life in chronic eye disease patients
Source: Sci Rep. 2022 Feb 10;12:2313. doi: 10.1038/s41598-022-06252-1 (PMC8831583; doi:10.1038/s41598-022-06252-1)
Supplement: Supplementary file 1 — Supplementary Information. [file 41598_2022_6252_MOESM1_ESM.docx]

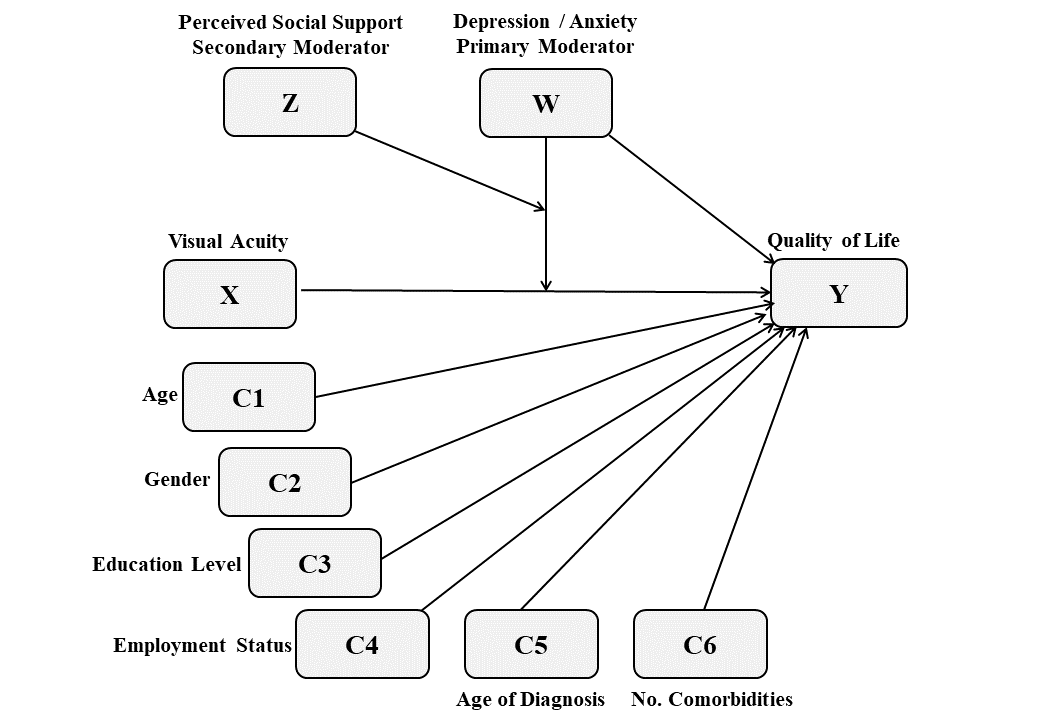


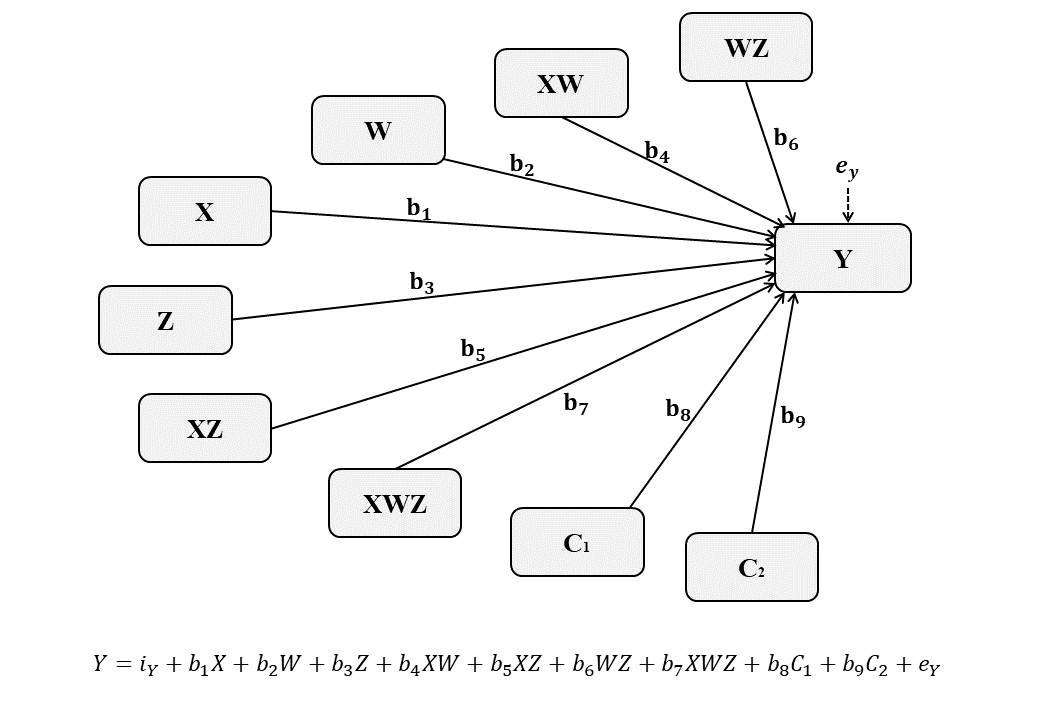


**Supplementary Figure 1**- Moderation model with perceived social support as secondary moderator (Z) and anxiety or depression and primary moderator (W) of the relationship between visual acuity (X) and health-related quality of life (Y).

| N=71 | |  | Coefficient | SE | t | *p* |
| --- | --- | --- | --- | --- | --- | --- |
| Model 1 - Perceived Social Support as secondary moderator for Depression    R^2^ = 0.39, MSE = 0.03  R^2^ Change = 0.00 | Constant | i_y_ | 0.78 | 0.16 | 4.86 | <0.001 |
|  | Visual Acuity (X) | b_1_ | -0.24 | 0.08 | -3.05 | 0.003 |
|  | Depression (W) | b_2_ | -0.03 | 0.03 | -1.04 | 0.302 |
|  | XW | b_3_ | -0.07 | 0.09 | -0.73 | 0.469 |
|  | Perceived Social Support (Z) | b_4_ | 0.01 | 0.04 | 0.38 | 0.707 |
|  | XZ | b_5_ | 0.11 | 0.14 | 0.77 | 0.445 |
|  | WZ | b_6_ | -0.05 | 0.05 | -1.05 | 0.298 |
|  | XWZ | b_7_ | 0.06 | 0.19 | 0.30 | 0.764 |
|  | Age (C1) | b_8_ | 0.00 | 0.00 | 0.37 | 0.715 |
|  | Gender (C2) | b_9_ | -0.05 | 0.05 | -0.98 | 0.332 |
|  | Education Level (C3) | b_10_ | 0.01 | 0.06 | 0.17 | 0.863 |
|  | Employment Status (C4) | b_11_ | -0.07 | 0.08 | -0.85 | 0.397 |
|  | Age of Diagnosis (C5) | b_12_ | 0.08 | 0.05 | 1.54 | 0.128 |
|  | Number of Comorbidities (C6) | b_13_ | -0.00 | 0.05 | -0.12 | 0.907 |
| Model 2 - Perceived Social Support as secondary moderator for Anxiety    R^2^ = 0.49, MSE = 0.02  R^2^ Change = 0.00 | Constant | i_y_ | 0.81 | 0.15 | 5.43 | <0.001 |
|  | Visual Acuity (X) | b_1_ | -0.29 | 0.06 | -4.59 | <0.001 |
|  | Anxiety (W) | b_2_ | -0.06 | 0.02 | -2.73 | 0.008 |
|  | XW | b_3_ | -0.16 | 0.07 | -2.35 | 0.022 |
|  | Perceived Social Support (Z) | b_4_ | -0.00 | 0.04 | -0.21 | 0.830 |
|  | XZ | b_5_ | 0.09 | 0.13 | 0.63 | 0.528 |
|  | WZ | b_6_ | -0.03 | 0.03 | -0.92 | 0.359 |
|  | XWZ | b_7_ | -0.07 | 0.13 | -0.51 | 0.614 |
|  | Age (C1) | b_8_ | -0.00 | 0.00 | -0.21 | 0.834 |
|  | Gender (C2) | b_9_ | -0.02 | 0.04 | -0.54 | 0.588 |
|  | Education Level (C3) | b_10_ | 0.03 | 0.06 | 0.54 | 0.593 |
|  | Employment Status (C4) | b_11_ | -0.01 | 0.08 | -0.17 | 0.836 |
|  | Age of Diagnosis (C5) | b_12_ | 0.07 | 0.05 | 1.58 | 0.119 |
|  | Number of Comorbidities (C6) | b_13_ | -0.01 | 0.05 | -0.28 | 0.777 |

**Supplementary Table 1** – Sensitivity analysis to examine the secondary moderated effect of perceived social support (Z), having anxiety or depression as primary moderator of the relationship between visual acuity (X) and health-related quality of life (Y).
